# Chromosome-Level Genome Assembly of the Speckled Blue Grouper (*Epinephelus cyanopodus*) Provides Insight into Its Adaptive Evolution

**DOI:** 10.3390/biology11121810

**Published:** 2022-12-13

**Authors:** Xiaoying Cao, Jiajun Zhang, Shunyun Deng, Shaoxiong Ding

**Affiliations:** 1 State Key Laboratory of Marine Environment Science, College of Ocean and Earth Sciences, Xiamen University, Xiamen 361000, China; 2 Function Laboratory for Marine Fisheries Science and Food Production Processes, Qingdao National Laboratory for Marine Science and Technology, Qingdao 266000, China

**Keywords:** *E. cyanopodus*, chromosomal assembly, genome annotation, immune response, adaptation

## Abstract

**Simple Summary:**

The coral reef-dwelling grouper *Epinephelus cyanopodus* has huge economic and ecological value. Due to its special reproductive strategy, complex social structure, and classification controversy, this species is a good model to study the coral reef ecosystem and the classification and speciation of groupers. The lack of genomic resources has hampered research into the genetic basis of their biological traits and adaptive evolution. Therefore, we have assembled a high-quality genome of *E. cyanopodus* and provided insights into the genetic basis of its adaptive evolution and rapid differentiation at the genomic level, as well as a foundation for subsequent studies on mechanisms of speciation, resistance breeding and genetic conservation for this species.

**Abstract:**

*Epinephelus cyanopodus* is a coral reef-dwelling grouper with important economic and ecological value and is widely distributed in the western Pacific Ocean. The lack of genomic resources for *E. cyanopodus* hinders its adaptive evolution and phylogeny research. We constructed the first high-quality genome of *E. cyanopodus* based on DNBSEQ, PacBio, and Hic sequencing technologies, with a genome size of 998.82 Mb, contig N50 of 5.855 Mb, and scaffold N50 of 41.98 Mb. More than 99.7% of contigs were anchored to 24 pseudochromosomes, and 94.2% of BUSCO genes were found in the *E. cyanopodus* genome, indicating a high genome assembly completeness. A total of 26,337 protein-coding genes were predicted, of which 98.77% were functionally annotated. Phylogenetic analysis showed that *E. cyanopodus* separated from its closely related species *Epinephelus akaara* about 11.5–26.5 million years ago, and the uplift of the Indo-Australian archipelago may have provided an opportunity for its rapid radiation. Moreover, several gene families associated with innate and adaptive immunity were significantly expanded in speckled blue grouper compared to other teleost genomes. Additionally, we identified several genes associated with immunity, growth and reproduction that are under positive selection in *E. cyanopodus* compared to other groupers, suggesting that *E. cyanopodus* has evolved broad adaptability in response to complex survival environment, which may provide the genetic basis for its rapid radiation. In brief, the high-quality reference genome of the speckled blue grouper provides a foundation for research on its biological traits and adaptive evolution and will be an important genetic tool to guide aquaculture and resolve its taxonomic controversies in future studies.

## 1. Introduction

Grouper (family Epinephelinae) is widely distributed in the tropical and subtropical seas of the Indian, Pacific, and Atlantic Oceans and is popular with consumers because of its bright body color and delicious flavor [1,2]. As typical coral reef fishes, groupers are rich in biodiversity, including 170 species in 16 genera [2,3,4,5,6]. Most of the species belong to the sympatric distribution, and the research on their origin, adaptive evolution, and rapid speciation have extremely high reference value for the formation and maintenance mechanisms of coral reef diversity [7]. However, only a few genome resources of economic groupers have been reported so far, which seriously hinders the study of the biology, adaptive evolution, and speciation of groupers.

The speckled blue grouper *Epinephelus cyanopodus* (Richardson, 1846) is a western Pacific species that inhabit coral reefs in lagoons or bays, feeding on sand-dwelling fishes and crustaceans [8]. The juveniles of *E. cyanopodus* are usually bluish-grey with faint dark dots and have yellow fins, while the yellow of adult fins gradually fades and disappears [2]. Owing to its variable body coloration and tender flesh, *E. cyanopodus* has become a popular delicious food and ornamental fish. Attempts to artificially breed speckled blue grouper have been made in many regions of China. Furthermore, *E. cyanopodus* is a good model for studies of coral reef ecosystems since they are protogynous hermaphroditic fish and top predators in the coral reef [2,9]. In addition, according to recent studies based on molecular markers of mitochondrial genes COI and ND2, it was proposed that *E. cyanopodus* and *Epinephelus flavocaeruleus* (Lacepède, 1802) may be synonymous. Therefore, *E. cyanopodus* is also a good model for studying the taxonomy and speciation of groupers. Owing to these factors, a high-quality genome of speckled blue grouper is not only an important genetic resource for adaptive evolution studies and resolving its taxonomic controversies, but will also provide a reference, and guidance for its biological characteristics and aquaculture.

Here we reported a high-quality reference genome of *E. cyanopodus* and provided insights into the hereditary basis of its rapid differentiation and adaptive evolution through comparative genomics analysis.

## 2. Materials and Methods

### 2.1. Sample Collection and Nucleic Acid Extraction

The sample of speckled blue grouper used for de novo genome sequencing and assembly was collected from Haikou (Hainan, China), and dissected after treatment with MS-222 (anesthetic tricaine methanesulfonate). DNA from fresh muscle tissue was extracted through the TIANamp Genomic DNA Kit (Tiangen, Beijing, China), and RNA from skin, fin and gill tissues were extracted using TRIzol reagent (Invitrogen, Carlsbad, CA, USA) for transcriptome sequencing according to the manufacturer’s instructions.

### 2.2. Library Construction and Genome Sequencing

For short-read sequencing, a pair-end library with a 300–500 bp insert size was successfully constructed and sequenced on the DNBSEQ-T7 platform (DNBSEQ^TM^ Technologies, Shenzhen, China), and the sequencing read length was 2 × 150 bp. For long-read sequencing, the PacBio CLR library was constructed based on genomic DNA and sequenced on the PacBio Sequel II platform (Pacific Biosciences, Menlo Park, CA, USA). For Hi-C sequencing, DNA isolated from muscle tissue was fixed with formaldehyde, then a Hi-C library was constructed and was also sequenced on the DNBSEQ-T7 platform. Additionally, RNA-seq libraries from three tissues were constructed and sequenced in the DNBSEQ-T7 platform.

### 2.3. Genome Assembly and Assessment

After filtering raw data for low quality, adapter sequences and reads containing more than 5% gap (N) by SOAPnuke software [10], the clean data were evaluated by NT alignment using BLAST [11] to exclude sample contamination. Subsequently, contigs were assembled by SOAPdenovo [12] using clean data and the genome size was estimated by K-mer analysis using Jellyfish [13] and GenomeScope [14] software. The sequencing data from PacBio Sequel II CLR libraries were further assembled using the MECAT2 [15] pipeline and polished using Pilon [16]. Purge_haplotigs are used to trim the assembly to reduce redundancy caused by heterozygosity. Lastly, contigs and scaffolds were anchored into chromosomes based on the Hic sequence reads through the Juicer (v1.5) [17] and 3D-DNA [18] workflow. To further improve the quality of the chromosome assembly, it was manually reviewed and refined using the Juicebox Assembly Tool (https://github.com/theaidenlab/juicebox, accessed on 5 January 2022).

In addition, genome quality was estimated by BUSCO3 [19] using the actinopterygii_odb9 database and by comparison reads of the small fragment library back to the assembled genome using BWA software.

### 2.4. Genome Annotation

For repetitive sequences, a repeat sequence library of *E. cyanopodus* was constructed by de novo and homology-based methods. In the de novo approaches, the *E. cyanopodus* repeat database was built by RepeatModeler [20], LTR_FINDER [21] and TRF v4.09 workflow, and RepeatMasker v4.0.6 [22] was used for classifying repeats. In the homology-based search, the RepeatProteinMask v3.3.0 (http://www.repeatmasker.org, accessed on 16 March 2022) and RepeatMasker v3.3.0 were used to detect repeat sequence and classify based on the Repbase library [23].

The structure annotation of protein-coding genes was performed through de novo prediction, homology prediction and RNA-seq-assisted methods. The Augustus v3.1 [24] was used for de novo prediction. For the homologous prediction, the amino acid sequences of *Larimichthys crocea*, *Takifugu rubripes*, *Oreochromis niloticus*, *Oryzias latipes*, *Epinephelus akaara*, *Epinephelus fuscoguttatus*, *Epinephelus lanceolatus*, *Epinephelus moara* and *Plectropomus leopardus* were loaded from NCBI and Ensemble database, and aligned to the genome of *E. cyanopodus* with genewise [25]. Furthermore, the protein-coding genes were further predicted by Stringtie and Transdecoder (http://transdecoder.github.io, accessed on 26 March 2022) methods based on transcripts from RNA-seq reads. Finally, the results were integrated through three evidence sets using the GLEAN pipeline [26]. The completeness of the gene sets was estimated using BUSCO software [19] and actinopterygii_odb9 was selected as the reference gene set.

For functional annotation, amino acid sequences obtained from gene structure prediction were aligned to known protein databases, including InterPro [27], Kyoto Encyclopedia of Genes and Genomes (KEGG, [28], Swissprot [29], gene ontology (GO, [30] and TrEMBL database [31], using Blastp program with a threshold value of E-value of 1 × 10^−5^.

### 2.5. Genome Synteny Analysis

The chromosomal synteny analysis of *E. cyanopodus* and red-spotted grouper *E. akaara* was performed by LASTZ (https://github.com/lastz/lastz (accessed on 15 April 2022); parameter: “-block_size = 2000”).

### 2.6. Comparative Genomic Analysis

We selected the annotated genes of *E. cyanopodus* and other 12 species, including six groupers (*E. akaara*, *E. fuscoguttatus*, *E. lanceolatus*, *E. moara*, *P. leopardus* and *Cromileptes altivelis*) and six other teleost species (*Lepisosteus oculatus*, *Gadus morhua*, *Danio rerio*, *T. rubripes*, *O. latipes*, and *L. crocea*) to identify gene family by TREEFAM tool (http://www.treefam.org/, accessed on 18 May 2022) [32]. First, all amino acid sequences of the 13 species above-mentioned were aligned by Blastp with an E-value threshold of 1 × 10^−5^ to identify orthologous genes. Subsequently, the single-copy genes shared from the 13 species were aligned using muscle v3.8.31 [33] and an ML phylogenetic tree was constructed with raxml v8.2.4 [34]. The divergence time among the 13 species was estimated through mcmctree in paml v4.7a [35]. Four calibration points (*L. oculatus* vs. *D. rerio* 295–334 Mya; *G. morhua* vs. *O. latipes* 141–166 Mya; *O. latipes* vs. *L. crocea* 105–145 Mya; *P. leopardus* vs. *E. lanceolatus* 26–94 Mya) from the TimeTree database (http://timetree.org/, accessed on 25 May 2022) were set to calibrate divergence time. In addition, to better understand the evolutionary dynamics of genes, gene family expansion and contraction analysis was performed using café v3.1 software [36] with the phylogenetic tree constructed above. Based on the results of the expansion, further enrichment analysis was performed using the GO and KEGG databases.

### 2.7. Identification of Positive Selection Genes

To identify positive selection genes (PSGs), five closely related species (*E. akaara*, *E. fuscoguttatus*, *E. lanceolatus*, *E. moara* and *C. altivelis*) to *E. cyanopodus* were also selected for analysis. First, sequence alignment was performed using diamond software [37], and the reciprocal best hits (RBHs) of all species were extracted and then aligned using muscle software v3.8.31 [33]. Subsequently, conserved sequences of RBHs were extracted by Gblocks [38] (parameters: -t= c -b1=4 -b2=5 -b3=8 -b4=2 -b5=a) and genes containing stop codons as well as non-triplet codons were filtered. Finally, the lineage-specific evolutionary rate of each branch was estimated using the Codeml program in the Paml v4.8 package [35]. A phylogenetic tree was constructed using the one-to-one genes above extracted. Branch-site models were used to detect PSGs, and the speckled blue grouper lineages were designated as foreground branches and subjected to a likelihood ratio test (LRT) to check whether a branch-site model containing positively selected codons (model = 2, NSsites = 2, fix_omega = 0, omega = 1.5) was more appropriate than a null model (model = 2, NSsites = 2, fix_omega = 1, omega = 1) that included only neutral or negative selection. *p*-values for model comparisons were calculated based on chi-square statistics, with *p* < 0.05 considered as a positive selection. Based on GO and KEGG annotations, we further performed the functional enrichment analysis (*p* < 0.05 by Fisher’s exact test) of positively selected genes. Pathways with an FDR cutoff of less than 0.05 were defined as significantly enriched pathways.

## 3. Results and Discussion

### 3.1. Genome Assembly and Evaluation

**Figure 1:**
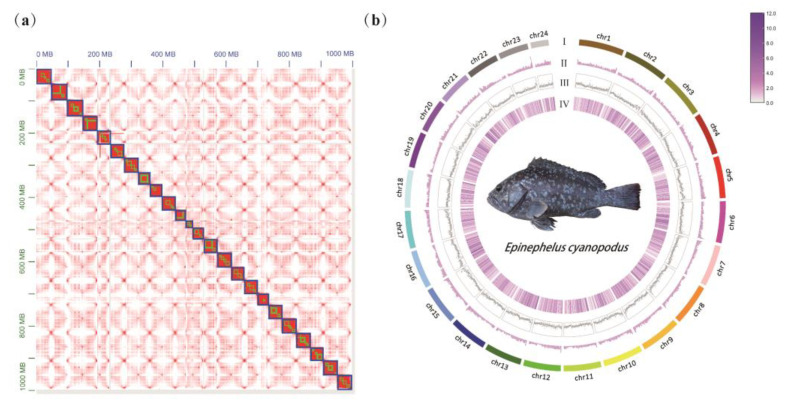
**Genomic information visualization of *E. cyanopodus*: *(*a**) Hi-C heatmap of *E. cyanopodus* genome assembly; and (**b**) genome characteristics of *E. cyanopodus*: (I) represents chromosome; (II) represents GC content; (III) represents the density of repeat sequence on each chromosome, and (IV) represents gene density on the chromosome.

A total of 174.34 Gb Raw reads of WGS sequencing data were obtained by the DNBSEQ T7 platform and were filtered by SOAPnuke software to obtain 172.39 Gb clean reads (Table S1). The clean reads were aligned to the NT database, indicating no exogenous contamination of the samples (Table S2). The genome size of *E. cyanopodus* was inferred to be 976.13 Mb with a heterozygosity of 0.309% and a GC content of 41.13% by K-mer analysis using WGS sequencing data (Table S3; Figure S1). A total of 155.77 Gb of raw data was obtained for de novo assembly based on the PacBio sequencing platform (Table S4), and 458 contigs with an N50 of 5.855 Mb and the longest read length of 31.6 Mb were constructed (Table 1). Furthermore, 196 Gb reads from the Hic library were obtained to help anchor the contig to the chromosomes (Table S5). About 99.7% of the contigs were anchored to 24 pseudochromosomes (chr), resulting in a 998.82 Mb genome with a Scaffold N50 of 41.98 Mb and the longest Scaffold length of 50.36 Mb (Figure 1a,b, Table 1 and Table S6). The genome size of the speckled blue grouper is slightly larger than that of the leopard coral grouper *P. leopardus* (784.57 Mb) [39], but slightly smaller than that of other fish in the *Epinephelus*, such as the brown-marbled grouper *E. fuscoguttatus* (1047 Mb) [40], red-spotted grouper *E. akaara* (1.135 Gb) [41].

The genome of *E. cyanopodus* has a higher quality assembly level, with Contig N50 values greater than most fish species, such as giant grouper *E. lanceolatus* (119.9 Kb) [42], the kelp grouper *E. moara* (2.22 Mb) [43] and *Astyanax mexicanus* (1.7 Mb) [44]. In addition, the high completeness (BUSCO 95.8%), high mapping rate (99.81%) and high coverage rate (99.79%) of short reads aligned to the genome also indicated the high assembly quality of the genome in speckled blue grouper (Table 1, Table S7 and Table S8).

### 3.2. Genome Annotation

Repetitive sequences were identified by de novo prediction and a homology search. A total of 391,109,130 bp repeat sequences were predicted, accounting for 39.157% of the genome (Table S9). Of these, transposable elements (TEs) were the most abundant, accounting for 36.706% of the genome. DNA transposons were dominated in TEs with the proportion of 17.022% genome assembly, followed by long interspersed elements (LINEs) 14.120% and long terminal repeats (LTRs) 7.022% of the genome (Table S10).

A total of 26,337 protein-coding genes were identified based on the genome with repetitive elements masked through de novo prediction, homology prediction and RNA-seq-assisted methods (Table 2). The average length of gene and CDS in *E. cyanopodus* were 17,793.4 bp and 1648.06 bp, respectively, while the average length of intron and exon were 1937.65 bp and 176.60 bp, respectively (Table 2). In addition, we compared the length distribution of the genes, CDS, exon, intron, and exon number between *E. cyanopodus* and two fish species (*O. niloticus, O. latipes*) (Figure S2, Table S11), and the results showed high consistency with the distribution feature of the genes among them, suggesting that the protein-coding genes were conserved in evolution in teleost fishes. Furthermore, a total of 26,013 (98.77%) predicted genes were functionally annotated with at least one of the SwissPort, NR, KEGG and GO databases (Table S12). Among them, a total of 22,065 (84.8%) genes were annotated with all the databases (Table S12), indicating a highly credible gene set. The completeness of the gene set was assessed using BUSCOs and actinopterygii_odb9 was selected as the reference gene set, of which 94.2% of complete BUSCOs were successfully identified (Table 1).

In addition, A total of 3898 noncoding RNA (ncRNA), including 1181 rRNAs, 1788 tRNAs, 473 snRNAs, and 466 miRNAs, were identified in the genome of *E. cyanopodus* (Table 3).

### 3.3. Chromosome Synteny Analysis

**Figure 2:**
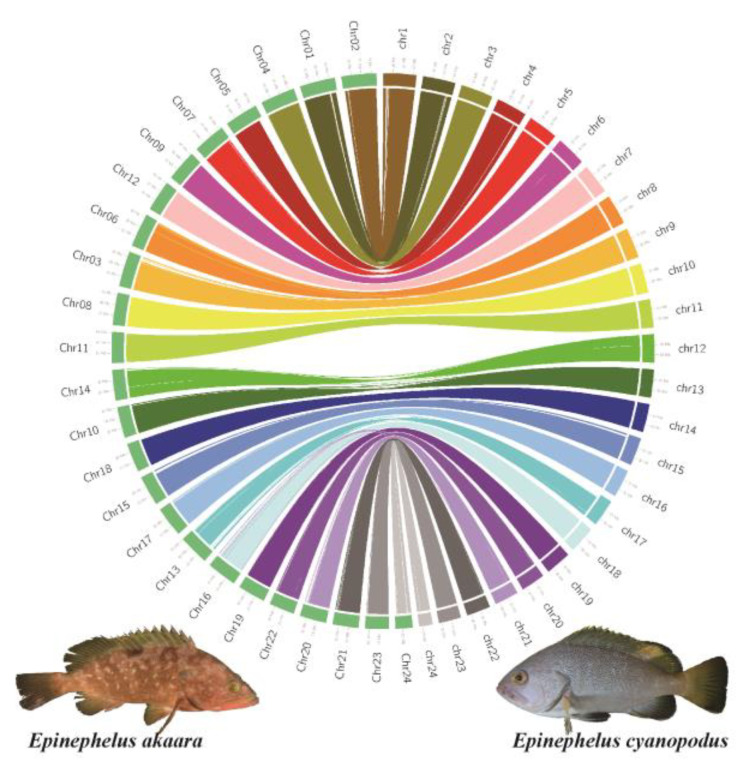
Genomic synteny analysis between *E. cyanopodus* and *E. akaara*. Each colored arc depicts inter-chromosomal synteny.

**Figure 3:**
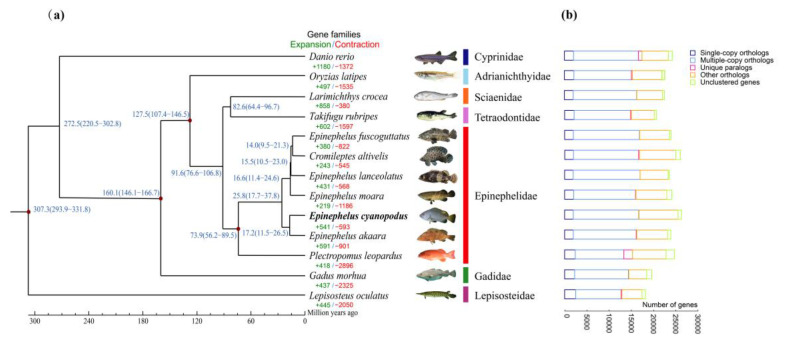
**Comparative genomic analysis visualization of *E. cyanopodus* and 12 teleost fishes:** (**a**) phylogenetic tree and divergence time constructed for 13 selected species. The blue numbers on the branches indicate the estimated divergence times (million years ago; confidence intervals 95%). The number represents the number of expanded (green) and contracted (red) gene families; and (**b**) comparison of gene family clusters. The horizontal axis display genes number and the vertical axis display species.

In addition, the chromosomes of *E. cyanopodus* and *E. akaara* have one-to-one pairwise collinearity with no fusion and fission events (Figure 2) and also indicate that the two species are more closely related genetically, consistent with the phylogenetic relationship (Figure 3a).

### 3.4. Divergence Time Estimation

A comparative genomics analysis was performed to infer the evolutionary history of *E. cyanopodus*. Clustering of gene families from 13 species yielded a total of 14,026 gene families, of which 1356 were single-copy gene families shared by all species (Figure 3b). Using single-copy genes shared by the species to construct a phylogenetic tree and estimate divergence time, the results showed that *E. cyanopodus* is most closely related to *E. akaara*; these two species were separated between 11.5 and 26.5 million years ago (mid-Miocene; Figure 3a). The *E. cyanopodus* is mainly distributed in the central Indo-Pacific Ocean [2], and its divergence time coincides with the time of the uplift of the Indo-Australian Archipelago in the Early Miocene (~23 Ma) [45]. The uplift of the archipelago and the emergence of a large number of new ecological niches provided the driving factors for its differentiation [7]. Similarly, the species of the genus *Epinephelus* and *Cromileptes* in this study were both differentiated in the mid-Miocene [7], which further indicates that the uplift of the Indo-Australian archipelago in the early Miocene and the decline of sea levels provided sufficient opportunities for adaptive radiation and diversification in groupers.

### 3.5. Gene Family Expansion

**Figure 4:**
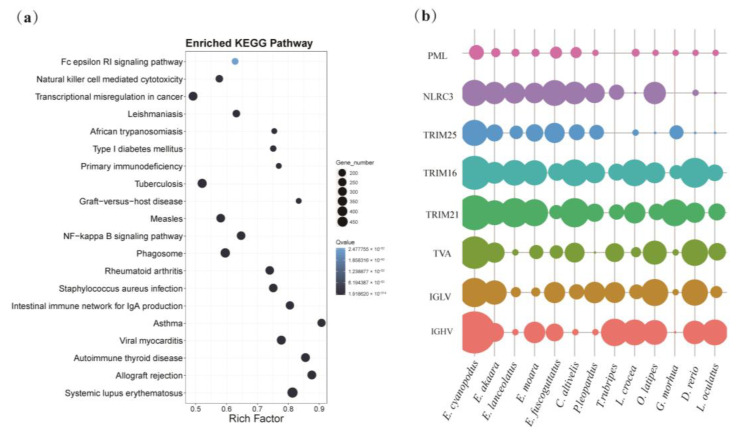
**Visualization of expanded gene families in *E. cyanopodus*:** (**a**) the top 20 significant enriched KEGG terms for expanded gene families in *E. cyanopodus* genome; and (**b**) the most significant expanded gene families in *E. cyanopodus*; the size of spots displays the number of genes.

Gene family expansions may play an important role in promoting phenotypic diversification and the evolution of environmental adaptations [46]. To better understand the evolutionary dynamics of genes, gene family expansion and contraction analysis was performed using café v3.1 software [36]. We identified 541 expanded gene families and 593 contracted gene families (*p* < 0.5) in speckled blue grouper by comparing gene families from 13 species. Based on the expansions of results, further enrichment analysis was performed using the GO and KEGG databases. The expanded gene family is mainly involved in the immune system, ion binding, endocrine system, digestive system, nervous system, sensory system, development, and environmental adaptation (Tables S13 and S14), revealing the adaptability of *E. cyanopodus* to complex living environments and resistance to multiple stresses. In particular, the immune system is significantly expanded, with all the signaling pathways of KEGG top20 concentrated in the immune system (Figure 4a).

Innate immunity is the internal barrier of fish to resist various exogenous pathogens, and plays an important role in their survival [47]. Nod-like receptors (NLR) are intrinsic innate immune molecules that are distributed on the surface or inside the membrane of immune cells, recognize pathogen-associated molecular patterns (PAMPs), and are widely involved in the recognition of pathogenic microorganisms and inflammatory responses [48]. *NLRC3*, a member of the NLR family, is significantly expanded in *E. cyanopodus* (Figure 4b) and has been shown to play an important role in resistance to various bacteria and viruses [49]. In addition, members of tripartite motif-containing (TRIM) family proteins are also significantly expanded in *E. cyanopodus*, such as promyelocytic leukemia *PML* (also known as *TRIM19*), *TRIM16*, *TRIM21* and *TRIM25* (Figure 4b). TRIM proteins play a key role in antiviral and mediating innate immune receptor-triggered signaling pathways by, for example, enhancing or inhibiting innate immune signaling in the antifungal, antiviral type I interferon, pro-inflammatory NF-kB and inflammasome pathways [50]. *NLRC3* and *TRIM* genes were significantly expanded in speckled blue grouper, implying an enhanced innate immunity.

Immunoglobulin (Ig), as a key effector of humoral immunity, can specifically recognize and neutralize antigens [51], and consists of two identical immunoglobulin heavy chains (IgH) and two identical immunoglobulin light chains (IgL). In response to the complex and changeable aquatic environment, immunoglobulins have evolved rich diversity, which is mainly reflected in the diversity of variable (V) and constant (C) regions of heavy and light chains. The V region of Ig (IGHV and IGLV) is responsible for recognizing and binding antigens, and the higher the diversity of the V region, the greater the ability to recognize and bind antigens [52]. T cells, a key factor of cellular immunity, have a diversity of receptors (T cell receptor, TCR), which enables them to recognize a large number of antigens. The diversity of TCR mainly depends on the difference of its variable region (TVA) and different recombination mechanisms [53]. Therefore, the expansion of IGHV, IGLV and TVA gene fragments in *E. cyanopodus* increases the random recombination diversity of Ig and TCR (Figure 4b), enabling it to specifically recognize a wider range of antigens. In summary, to cope with the complex coral reef environment, *E. cyanopodus* have improved their innate immunity along with adaptive immunity.

### 3.6. Identification of Positively Selected Genes

To understand the molecular basis of rapid differentiation and adaptation to the environment in *E. cyanopodus*, we identify its positively selected genes (PSGs) using Paml, showing that a total of 1652 PSGs (*p* < 0.05) were identified (see Supplemental Data). The enrichment results suggest that PSGs sets may be involved in the regulation of immune response, growth, reproduction, cell migration and differentiation, and circadian rhythm, etc. (Tables S15 and S16), suggesting that the speckled blue grouper has undergone extensive adaptation, providing a reliable genetic basis for its rapid radiation.

Several genes associated with innate immunity were found to be positively selected in the speckled blue grouper lineage, such as complement component (*C3*, *C5*, *C6*), complement factor (*CFB*, *CFI*), C-X-C motif chemokine 10 (*CXCL10*). In contrast to the imperfect adaptive immune mechanisms, fishes rely mainly on innate immunity to fight various pathogenic microorganisms. The complement system is an important part of innate immunity. As the hub of the three activation pathways of the complement system (classical pathway, lectin pathway and alternative pathway), the *C3* component must be activated to realize a cascade of complement response to achieve its effects [54,55]. *C5* can be cleaved by *C5* convertase to *C5a* and *C5b*, which then form a membrane attack complex (MAC) with *C6*, *C7*, *C8* and *C9*, eventually causing the foreign pathogen cells to be dissolved and broken [54]. *CFB* and *CFI* play a crucial role in the alternative pathway of complement [55,56], while *CXCL10* plays an important function in the inflammatory response of innate immunity [57]. Moreover, we also identified several positively selected genes for immune-related cytokines, such as Interleukin-12 subunit beta (*Il12b*), Interleukin-17 receptor E (*Il17re*), and TNF receptor-associated factor 3 (*Traf3*). Interleukin can activate all kinds of immune cells in time to respond to external stimuli and is responsible for signaling between immune cells [58]. *Traf3* is not only involved in immune signaling but also essential for the proliferation and survival of immune cells [59]. The evolutionary changes in immune genes suggest that the immunity and disease resistance of the speckled blue grouper are enhanced compared to other groupers, as *E. cyanopodus* are adapted to a wider range of water depths (range 2–150 m) and mainly around the top of isolated coral reefs in lagoons or bays [2],where they need to cope with a greater abundance of pathogenic microorganisms in their habitat, whereas the other groupers in this study had a narrower range of adaptation to water depths and mainly inhabit in the middle and lower layers [2].

In addition, we also identified positive selection genes associated with cholesterol synthesis and metabolism (*LSS*, *NSDHL*, *LICH*, *ERG1*, *EBP*) and insulin secretion (*PRKCA*, *PRKCB*, *KCNN3*, *PACAPRI*, *CACNA1C*, *CACNA1F*, *RIMS2*, *CREB3L4*) in the speckled blue grouper lineage. Studies have shown that cholesterol anabolism plays an important role in fish growth [60,61], while insulin secretion can also increase susceptibility to fish hunger, which leads to increased food intake and ultimately promotes growth [62]. Several PSGs in Ras and Rho protein signal transduction, Ras and Rho GTPase binding, and Rho guanyl-nucleotide exchange factor activity were also identified, which are critical to the regulation of cell growth, proliferation, differentiation and migration [63,64]. Furthermore, we also found that several genes of the GnRH signaling pathway (*GnRHR-II*, *ADCY1*, *ADCY2*, *ADCY5*, *ADCY9*, *PLCB1*, *IP3R2*) were positively selected, suggesting that evolutionary changes may contribute to the reproduction and gonadal development in *E. cyanopodus*.

## 4. Conclusions

We assembled the first high-quality reference genome of the speckled blue grouper, with a genome size of 998.82 Mb and predicted 26,337 protein-coding genes. *E. cyanopodus* diverged approximately 11.5–26.5 million years ago (Ma) from its closely related species *E. akaara*, and the uplift of the Indo-Australian archipelago may have provided an opportunity for its rapid radiation. In addition, the expansion of innate and adaptive immunity genes, as well as the evolutionary changes of immunity, growth, and reproduction genes, suggest that *E. cyanopodus* have broad adaptability to the complex coral reef environment, which may have provided the genetic basis for its rapid radiation. This reference genome provides insights into the adaptive evolution and rapid radiation of *E. cyanopodus*, and will be an important genetic tool to resolve its taxonomic controversies and speciation mechanisms in future studies.

## Figures and Tables

**Table 1:** The statistics of the *Epinephelus cyanopodus* genome assembly and annotation.

Assembly and Annotation Metrics	Number or Percentag
Number of contigs	458
Total Length of Contig(bp)	998,645,601
Contigs N50 (bp)	5,850,290
Contigs N90 (bp)	1,343,332
Maximum length of contig (bp)	31,638,094
GC content (%)	41.13%
Scaffold N50 (bp)	41,979,993
Scaffold N90 (bp)	36,023,923
Hi-C anchored ratio	99.70%
Gene number	26,337
**Genome BUSCO**	
Complete BUSCOs (C)	95.8%
Complete and single-copy BUSCOs (S)	93.4%
Complete and duplicated BUSCOs (D)	2.4%
Fragmented BUSCOs (F)	2.1%
Missing BUSCOs (M)	2.1%
**Gene set BUSCO**	
Complete BUSCOs (C)	94.20%
Complete and single-copy BUSCOs (S)	90.40%
Complete and duplicated BUSCOs (D)	3.80%
Fragmented BUSCOs (F)	4.00%
Missing BUSCOs (M)	1.80%

**Table 2:** Summary statistics of predicted protein-coding genes in *E. cyanopodus*.

	Gene Set	Gene Numbers	Gene Length (bp)	CDS Length (bp)	Intron Length (bp)	Exon Length (bp)	Exons per Gene
**De novo**	Augustus	35,837	13,973.06	1299.72	1980.92	176	7.4
**Homolog**	*Larimichthys crocea*	23,855	25,019.98	1660.45	2792.69	177	9.36
	*Takifugu rubripes*	21,356	28,138.59	1592.36	3365.60	179	8.89
	*Oreochromis niloticus*	28,135	48,902.73	1654.82	6417.90	198	8.36
	*Oryzias latipes*	22,967	32,996.88	1665.46	4012.74	189	8.81
	*Epinephelus akaara*	26,107	23,122.33	1708.31	2295.96	165	10.33
	*Epinephelus fuscoguttatus*	26,333	21,472.99	1684.42	2248.34	172	9.8
	*Epinephelus lanceolatus*	26,129	20,700.34	1738.50	2138.27	176	9.87
	*Epinephelus moara*	26,847	19,858.37	1593.64	2229.24	173	9.19
	*Plectropomus leopardus*	26,178	79,013.83	1656.29	9731.50	185	8.95
**Transcript**	Stringtie & Transdecoder	50,143	20,453.71	1045.25	499.54	230	4.55
	**GLEAN**	26,337	17,793.40	1648.06	1937.65	177	9.33

**Table 3:** Summary statistics of noncoding RNA in *E. cyanopodus*.

Type		Copy Number	Average	Total	% of
			Length(bp)	Length(bp)	Genome
rRNA	rRNA	1181	136.57	161,293	0.0161
	18S	180	154.94	27,890	0.0028
	28S	199	206.02	40,997	0.0041
	5.8S	11	107.27	1180	0.0001
	5S	791	115.33	91,226	0.0091
snRNA	snRNA	473	134	63,380	0.0063
	CD-box	130	103.88	13,505	0.0014
	HACA-box	79	151.05	11,933	0.0012
	splicing	256	141.15	36,134	0.0036
miRNA		466	85.17	39,687	0.004
tRNA		1778	75.74	134,663	0.0135

## Data Availability

The whole-genome project of *E. cyanopodus* was deposited at NCBI (PRJNA844481), and the genome data was deposited in National Center for Biotechnology Information (NCBI), and the following accession numbers: JAMWDX000000000.

## Supplementary Materials

The following supporting information can be downloaded at: https://www.mdpi.com/article/10.3390/biology11121810/s1, Figure S1: K-mer distribution of the speckled blue grouper with a k-mer size of 17; Figure S2: Comparison of gene structure between *E. cyanopodus* and other species; Table S1: The statistics of WGS sequencing data; Table S2: The alignment of clean reads with NR database; Table S3: Results of k-mer analysis of the genome; Table S4: Statistics of PacBio sequencing results; Table S5: statistics of Hi-C sequencing results; Table S6: The characteristics of pseudochr of *E. cyanopodus*; Table S7: Coverage statistics of genome reads; Table S8: The statistics of genome SNP; Table S9: Summary statistical of repeated sequences; Table S10: Summary statistics of transposable elements (TEs); Table S11: Comparison of gene sets in *E. cyanopodus* and other fishes; Table S12: Function annotation of protein coding genes of *E. cyanopodus*; Table S13: KEGG enrichment analysis of significantly expanded gene families in *E. cyanopodus*; Table S14: GO enrichment analysis of significantly expanded gene families in *E. cyanopodus*; Table S15: KEGG enrichment analysis of positive selection genes (PSGs) in *E. cyanopodus*; Table S16: GO enrichment analysis of positive selection genes (PSGs) in *E. cyanopodus*. Supplemental Data: The alignment sequence of PSGs.
