# Supplementary material for: Chromosome-Level Genome Assembly of the Speckled Blue Grouper (Epinephelus cyanopodus) Provides Insight into Its Adaptive Evolution
Source: Biology (Basel). 2022 Dec 13;11(12):1810. doi: 10.3390/biology11121810 (PMC9775623; doi:10.3390/biology11121810)
Supplement: Supplementary file 1 [file biology-11-01810-s001.zip › Supplemental file.pdf]

Supplementary Material for:

Chromosome-level genome assembly of the speckled blue grouper (*Epinephelus cyanopodus*) provides insight into its adaptive evolution

Xiaoying Cao[1], Jiajun Zhang[1], Shunyun Deng[1], Shaoxiong Ding[1, 2, *]

[1]Xiamen State Key Laboratory of Marine Environment Science, College of Ocean and Earth Sciences, Xiamen University, Xiamen 361000, China

[2]Function Laboratory for Marine Fisheries Science and Food Production Processes, Qingdao National Laboratory for Marine Science and Technology, Qingdao 266000, China

[*]Correspondence: sxding@xmu.edu.cn

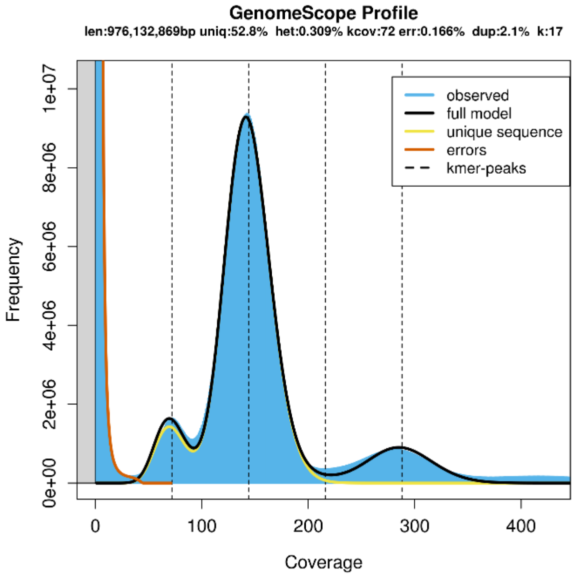

Figure S1 K-mer distribution of the speckled blue grouper with a k-mer size of 17

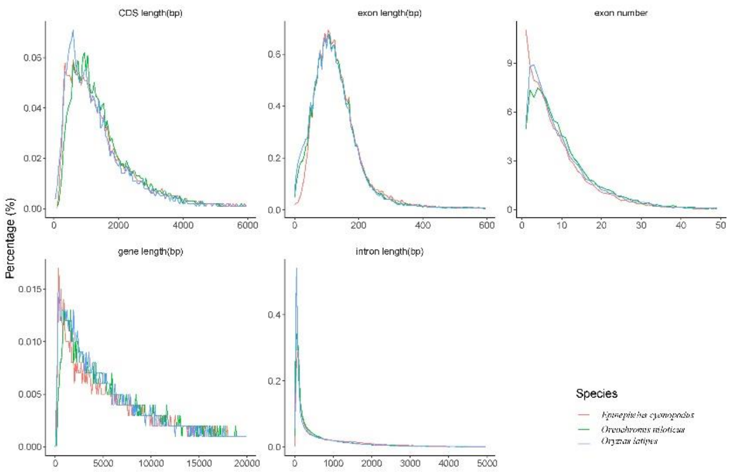

Figure S2 Comparison of gene structure between *E. cyanopodus* and other species

Table S1 The statistics of WGS sequencing data

Raw reads (M)	Clean reads (M)	Clean bases (Gb)	Q20 (%)	Q30 (%)	Ratio (%)
1743.4	1723.9	172.39	98.03	93.7	98.88

Table S2 The alignment of clean reads with NR database

**Rank**	**Species**	**Number**	**Percentage (%)**
1	*Larimichthys crocea*	165	1.65
2	*Amphiprion ocellaris*	56	0.56
3	*Seriola dumerili*	55	0.55
4	*Lates calcarifer*	53	0.53
5	*Stegastes partitus*	44	0.44
6	*Seriola lalandi*	42	0.42

Table S3 Results of k-mer analysis of the genome

**K-mer**	**Genome Length**	**Repeat Length**	**Unique Length**	**Heterozygosity**
17	976,132,869	461,192,649	514,940,221	0.31%

Table S4 Statistics of PacBio sequencing results

**Total Number**	**Total Length(bp)**	**N50 (bp)**	**N90 (bp)**	**Max Length(bp)**
7,021,700	155,766,171,228	35,344	12,206	257,569

Table S5 Statistics of Hi-C sequencing results

	**Reads 1**	**Reads 2**
Total Reads	981,775,584	981,775,584
Mapped Reads	932,174,162	891,636,134
Mapping Ratio	94.95%	90.82%

Table S6 The characteristics of pseudochr of *E. cyanopodus*

Chromosome ID	Length (bp)	Percentage
1	50,363,113	5.06%
2	49,546,324	4.98%
3	48,902,940	4.91%
4	45,868,825	4.61%
5	45,176,134	4.54%
6	45,071,231	4.53%
7	44,778,239	4.50%
8	44,686,160	4.49%
9	44,671,015	4.49%
10	44,435,251	4.46%
11	41,979,993	4.22%
12	41,708,438	4.19%
13	41,656,757	4.18%
14	41,599,946	4.18%
15	41,559,270	4.17%
16	41,547,119	4.17%
17	40,671,820	4.08%
18	40,483,175	4.07%
19	38,838,155	3.90%
20	36,149,711	3.63%
21	36,023,923	3.62%
22	35,995,713	3.61%
23	33,670,366	3.38%
24	20,405,733	2.05%
Total	995,789,351	99.70%
Unmap	3,032,250	0.30%

Table S7 Coverage statistics of genome reads

Items	Result
Reads mapping rate (%)	99.81
Genome average sequencing depth (×)	171.06
Coverage of genome (%)	99.79
Coverage of genome > 4× (%)	99.68
Coverage of genome > 10× (%)	99.52
Coverage of genome > 20× (%)	99.27

Table S8 The statistics of genome SNP

Items	Number	Percentage(%)
All SNP	1,435,304	0.17
Heterozygosis SNP	1,413,323	0.167
Homozygosis SNP	21,874	2.58E-03

Table S9 Summary statistical of repeated sequences

Type	Repeat Size(bp)	% of genome
TRF	35,006,529	3.505
RepeatMasker	95,542,772	9.566
RepeatProteinMask	35,818,559	3.586
De novo	360,802,032	36.123
Total	391,109,130	39.157

Table S10 Summary statistics of transposable elements (TEs)

Type	Repbase TEs		Protein TEs		*De novo* TEs		Combined TEs	
	Length (bp)	% of genome	Length (bp)	% of genome	Length (bp)	% of genome	Length (bp)	% of genome
DNA	50,916,126	5.098	954,920	0.096	146,124,735	14.63	170,023,344	17.022
LINE	34,099,727	3.414	28,911,341	2.895	124,819,458	12.497	141,036,944	14.12
SINE	5,146,947	0.515	0	0	4,703,023	0.471	9,015,117	0.903
LTR	12,684,555	1.27	5,959,783	0.597	63,583,004	6.366	70,141,879	7.022
Others	12,607	0.001	0	0	0	0	12,607	0.001
Unknown	0	0	0	0	47,963,183	4.802	47,963,183	4.802
Total	95,542,772	9.566	35,818,559	3.586	344,362,483	34.477	366,631,196	36.706

Table S11 Comparison of gene sets in *E. cyanopodus* and other fishes

Species	Gene numbers	Gene length (bp)	CDS length (bp)	Intron length (bp)	Exon length (bp)	Exons per gene
Epinephelus cyanopodus	26,337	17,793.40	1,648.06	1,937.65	176.60	9.33
Oreochromis niloticus	21,437	14,903.11	1,714.22	1,332.07	157.25	10.90
Oryzias latipes	19,699	12,145.58	1,515.82	1,148.61	147.82	10.25

Table S12 Function annotation of protein coding genes of *E. cyanopodus*

Database	Number	Percentage
Total	26,337	100%
Swissprot-Annotated	23,876	91.04%
KEGG-Annotated	23,143	87.87%
TrEMBL-Annotated	26,000	98.72%
Interpro-Annotated	24,613	93.45%
GO-Annotated	18,398	69.86%
Overall	26,013	98.77%

Table S13 KEGG enrichment analysis of significantly expanded gene families in *E. cyanopodus*

Table S14 GO enrichment analysis of significantly expanded gene families in *E. cyanopodus*

Table S15 KEGG enrichment analysis of positive selection genes (PSGs) in *E. cyanopodus*

Table S16 GO enrichment analysis of positive selection genes (PSGs) in *E. cyanopodus*

Tables S13–S16 are listed as excel files due to their large size.
